# Bridging Learning in Medicine and Citizenship During the COVID-19 Pandemic: A Telehealth-Based Case Study

**DOI:** 10.2196/24795

**Published:** 2021-03-04

**Authors:** Thiago Cerqueira-Silva, Roberto Carreiro, Victor Nunes, Louran Passos, Bernardo F Canedo, Sofia Andrade, Pablo Ivan P Ramos, Ricardo Khouri, Carolina Barbosa Souza Santos, Jedson Dos Santos Nascimento, Aurea Angélica Paste, Ivan De Mattos Paiva Filho, Marília Santini-Oliveira, Álvaro Cruz, Manoel Barral-Netto, Viviane Boaventura

**Affiliations:** 1 Universidade Federal da Bahia Salvador Brazil; 2 Instituto Gonçalo Moniz Fundação Oswaldo Cruz Salvador Brazil; 3 Centro de Integração de Dados e Conhecimentos para Saúde-CIDACS Fundação Oswaldo Cruz Salvador Brazil; 4 Hospital Santa Izabel Salvador Brazil; 5 Universidade do Estado da Bahia Salvador Brazil; 6 Fundação ProAR Salvador Brazil; 7 Universidade Salvador - UNIFACS Salvador Brazil; 8 Secretaria Municipal de Saúde de Salvador Salvador Brazil; 9 Instituto Nacional de Infectologia Evandro Chagas Fundação Oswaldo Cruz Salvador Brazil

**Keywords:** medical education, surveillance, COVID-19, education, telehealth, training, impact, medical student, triage, epidemiology, monitoring

## Abstract

**Background:**

COVID-19 presented great challenges for not only those in the field of health care but also those undergoing medical training. The burden on health care services worldwide has limited the educational opportunities available for medical students due to social distancing requirements.

**Objective:**

In this paper, we describe a strategy that combines telehealth and medical training to mitigate the adverse effects of the COVID-19 pandemic.

**Methods:**

A toll-free telescreening service, Telecoronavirus, began operations in March 2020. This service was operated remotely by supervised medical students and was offered across all 417 municipalities (14.8 million inhabitants) in the Brazilian state of Bahia. Students recorded clinical and sociodemographic data by using a web-based application that was simultaneously accessed by medical volunteers for supervision purposes, as well as by state health authorities who conducted epidemiological surveillance and health management efforts. In parallel, students received up-to-date scientific information about COVID-19 via short educational videos prepared by professors. A continuously updated triage algorithm was conceived to provide consistent service.

**Results:**

The program operated for approximately 4 months, engaging 1396 medical students and 133 physicians. In total, 111,965 individuals residing in 343 municipalities used this service. Almost 70,000 individuals were advised to stay at home, and they received guidance to avoid disease transmission, potentially contributing to localized reductions in the spread of COVID-19. Additionally, the program promoted citizenship education for medical students, who were engaged in a real-life opportunity to fight the pandemic within their own communities. The objectives of the education, organization, and assistance domains of the Telecoronavirus program were successfully achieved according to the results of a web-based post-project survey that assessed physicians’ and students’ perceptions.

**Conclusions:**

In a prolonged pandemic scenario, a combination of remote tools and medical supervision via telehealth services may constitute a useful strategy for maintaining social distancing measures while preserving some practical aspects of medical education. A low-cost tool such as the Telecoronavirus program could be especially valuable in resource-limited health care scenarios, in addition to offering support for epidemiological surveillance actions.

## Introduction

SARS-CoV-2 emerged as a novel coronavirus in December 2019, linked to a cluster of respiratory diseases [1]. The pathogen was identified to be the causative agent of COVID-19, a disease that rapidly acquired the status of a pandemic with over 85.3 million cases reported globally as of January 2021, resulting in 1.85 million total deaths [1,2]. COVID-19 is characterized by fever, cough, and dyspnea, which can progress to severe pneumonia, requiring mechanical ventilation [3]. At the onset of the pandemic, in a scenario where no effective or safe antiviral treatments nor vaccines for SARS-CoV-2 infection were available [4,5], nonpharmacological interventions were central to COVID-19 mitigation strategies in most countries. The nonpharmacological interventions enforced included stay-at-home orders, strict lockdowns, contact tracing, and social distancing recommendations—all of which are evidence-based strategies designed to effectively reduce the rate of spread of SARS-CoV-2 [6]. However, one caveat of implementing social distancing is its potential to delay health assistance for patients with acute infection who fear seeking health care services [7,8]. In addition, policies designed to avoid crowding, limitations on the use of public transportation, and shortages in the availability of personal protective equipment restricted routine medical school activities during times of isolation [9].

Toward the end of March 2020, several European countries were already facing the collapse of health systems due to the scarcity of human and material resources amidst increased demand [10,11]. Additional emergency measures included recruiting senior medical students to perform frontline work to reduce the burden on formally trained health care workers [12]. The implementation of telemedicine solutions and automated risk screening solutions via web apps were also proposed as solutions to mitigate the pandemic [13].

To respond to the challenges presented by the COVID-19 pandemic, we proposed a telescreening solution, termed *Telecoronavirus*, to reduce the burden on the local public health system in Bahia and to limit the spread of disease. Medical students were invited to voluntarily participate in a task force that served the population of the Brazilian state of Bahia, consisting of 14.8 million individuals across 417 municipalities, with a territorial extension comparable to that of France. The Telecoronavirus program was operational as of March 24, 2020, that is, 18 days after the first COVID-19 case was confirmed in the state of Bahia. At that time, a total of 63 COVID-19 cases had been confirmed in 12 (2.9%) cities in the state of Bahia [14]. This paper details the strategy employed by the Telecoronavirus program, which can be adapted for use in future waves of COVID-19, as well as other epidemics.

## Methods

To provide screening assistance to the population during the pandemic and offer high-quality information regarding coronavirus symptoms, we engaged supervised medical students through a combination of telehealth services and medical training. The volunteer program, termed Telecoronavirus, involved students in their final three years of undergraduate activities and physicians, faculty members, and researchers. The program was also designed to (1) reduce the burden on the hospital and health services infrastructure by preventing patients with mild symptoms from making unnecessary visits and (2) limit the spread of the virus by reducing the circulation of patients with symptoms that did not warrant immediate medical attention.

We created an organizational structure capable of interfacing academic institutions, government and regulatory bodies, and civil society. The recruitment of volunteers (students and physicians) was performed remotely using an online educational platform. Students and physicians had their identities respectively confirmed through official lists provided by medical schools and the regional medical board of the state of Bahia (Conselho Regional de Medicina do Estado da Bahia). Recruited students were required to complete video-based classes with instructions on the program’s operational objectives and the screening algorithm employed, followed by a test to validate their understanding.

The Telecoronavirus screening algorithm was elaborated by a committee formed of members with expertise in pneumology, infectious diseases, emergency care, and telehealth, in addition to a representative from the regional medical board. A flowchart (Multimedia Appendix 1) was designed for volunteers to follow, instructing them to collect clinical data and provide general guidance on hygiene and social distancing by using colloquial language to aid users’ comprehension and to standardize the information provided by the service. 

**Figure 1:**
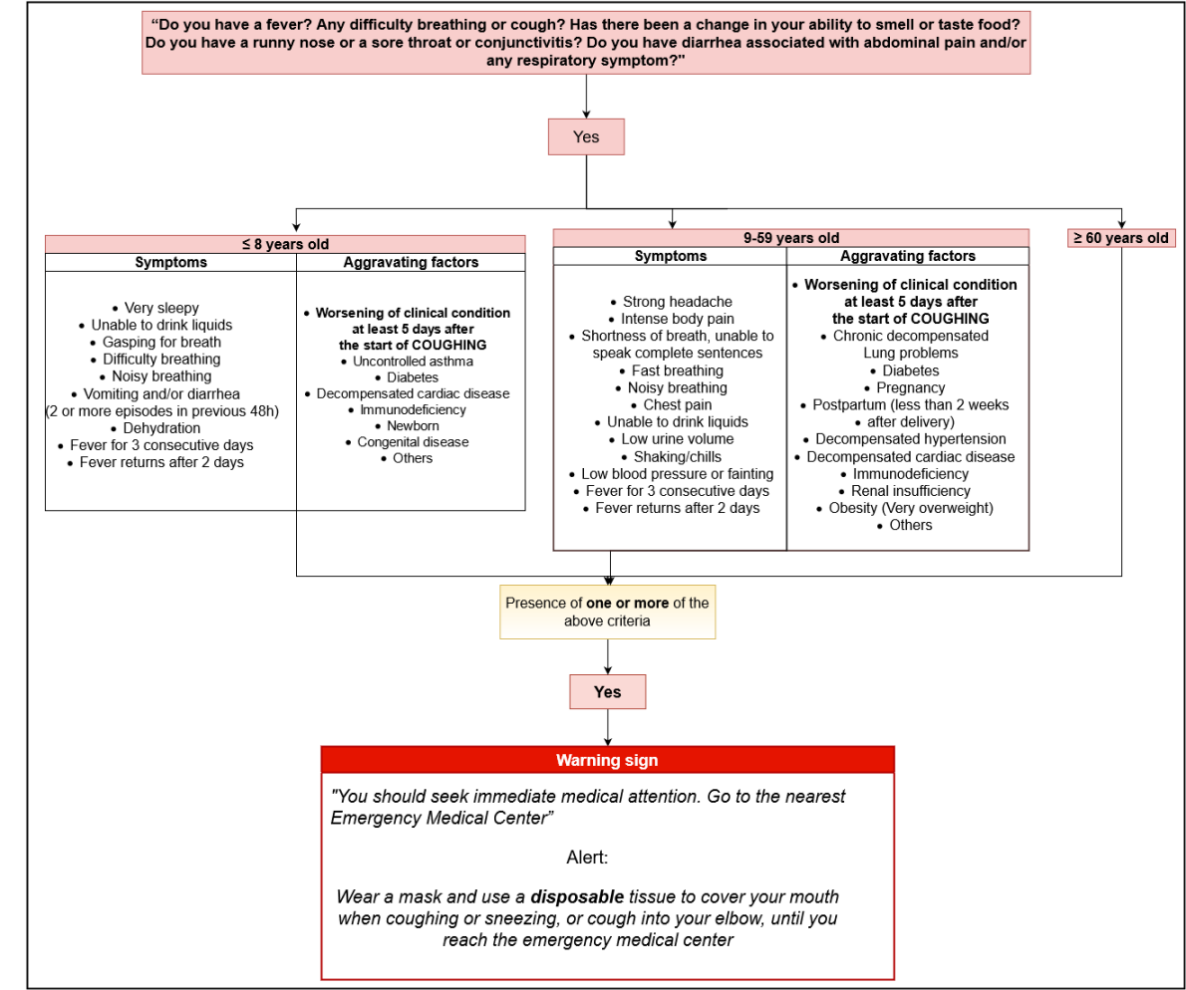
Summary of the Telecoronavirus flowchart used by volunteers, detailing clinical conditions that could lead to potentially poor outcomes (Bahia, Brazil).

The flowchart was constantly updated to reflect the most recent emerging scientific evidence on the novel coronavirus, undergoing 20 revisions during the project. Each version of the flowchart generated was identified with a date and link (plus a quick response or QR code), making it easy for all volunteers to access the most up-to-date version. Updated links were sent daily via a messaging application prior to commencing each shift at the service. Figure 1 provides a summary of the final version (number 20) of the flowchart, detailing clinical conditions that could lead to potentially poor outcomes.

In parallel, professors prepared short educational videos synthesizing up-to-date scientific evidence to improve students’ knowledge regarding COVID-19. In total, 20 videos were produced, exploring a range of diverse subjects such as epidemiology, clinical manifestations, special conditions (comorbidities and pregnancy), differential diagnoses, interpretation of diagnostic testing, airway management, psychological impacts, and socioethical aspects related to COVID-19.

A statewide toll-free number was set up, and operations were started on March 24, 2020. Call center employees redirected calls from potential patients with COVID-19 (ie, users) to the mobile phones of medical students who were remotely supervised by 133 physicians (residents or physician volunteers whose specialties were not directly applicable to frontline assistance). Two physicians supervised each group of 20 volunteers.

A custom web-based application was designed to record the users’ demographic and clinical data, including address, sex, age, symptoms, comorbidities, and other clinical conditions related to poor outcomes, as shown in Figure 1. Users with one or more of the clinical conditions described in the flowchart were advised to seek medical attention. The application incorporated tools to simultaneously allow data recording by the students, thereby allowing supervisors to examine the data input. The generated electronic database complied with ethical guidelines, and state health authorities were able to simultaneously access data for epidemiological surveillance purposes and aid the management of health resources. The total monthly cost for the Telecoronavirus program was about US $10,000, including the expenses for a call center service, publicity, and technical support.

At the end of each evaluation, users were advised to either stay at home or seek emergency medical assistance. All users received educational guidance on how to reduce the spread of the virus.

In order to obtain volunteers’ opinions about their experience with the Telecoronavirus program, a web-based post-project survey (Multimedia Appendix 2) was administered to assess physicians’ and students’ perceptions about the three domains of the Telecoronavirus program: education, organization, and assistance. The education domain included learning about COVID-19, telemedicine skills, telehealth skills (survey questions 6, 11-14). The organization domain comprised remote supervision of the telehealth service and flowchart quality (survey questions 4-8). The assistance domain addressed the perception that patients followed recommendations, the social impact of the Telecoronavirus service, social commitment and citizenship, and sense of accomplishment (survey questions 9, 10, and 14).

The survey aimed to evaluate the quality of service offered by the program, as well as its impact on technical education and citizenship learning. All volunteers were invited to submit their responses via the web-based survey. Survey responses are presented as mean and SD values, derived from a continuous rating scale ranging from 1 to 10, or as absolute values (expressed as n/N) with corresponding frequency (expressed as percentages) for categorical questions. Considering that this work entails an evaluation of public health service and that only anonymous data were collected via the surveys, the present case study was exempted from an ethics review in accordance with national research ethics guidelines (Resolution 466/12, Brazilian Health Council).

## Results

The Telecoronavirus program operated between March 24 and July 31, 2020. Users could contact the center via a statewide toll-free number from anywhere within the state of Bahia. In partnership with the state government of Bahia, the service was publicized across the state using billboards and SMS text messages, in addition to multiple interviews broadcast on various television channels. 

**Figure 2:**
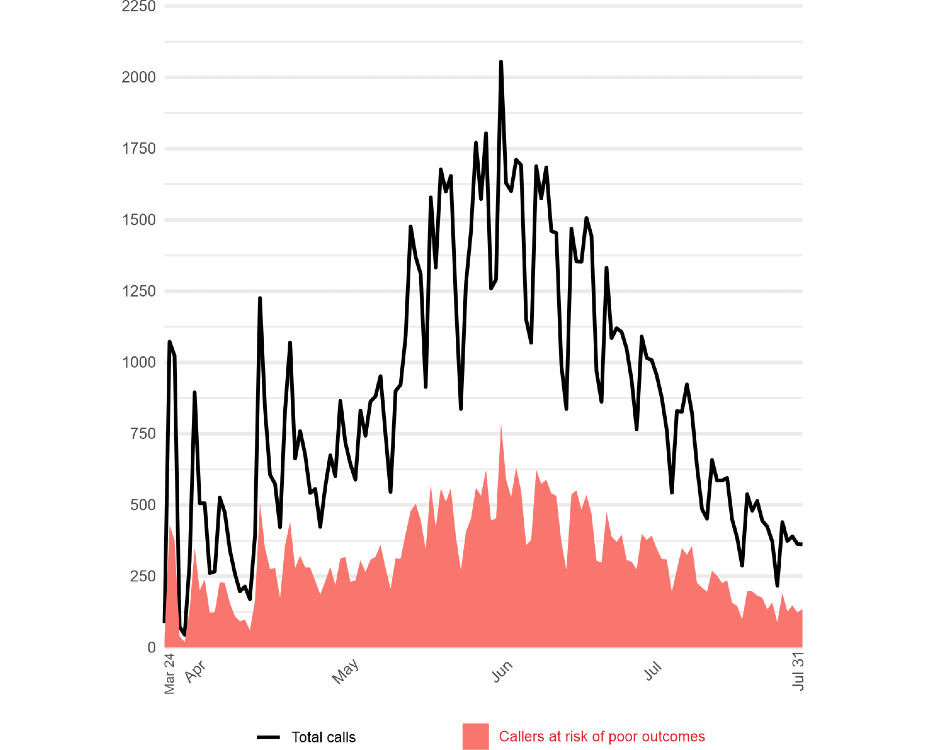
Total number of calls received daily by Telecoronavirus, including the numbers of callers deemed at risk of poor outcomes between March 24 and July 31, 2020 (Bahia, Brazil).

By July 31, that is, day 130 of commencing the operation, a total of 111,965 users who resided in 343 of the state’s municipalities (ie, more than 80%) had contacted the service. With respect to the daily call volume, the highest demand occurred between May 14 and June 10, with a peak of 2055 calls received on June 1 (Figure 2). The majority of users were women (67,626/111,965, 60.4%) aged between 20 and 59 years (90,243/111,965, 80.6%). On average, the consultation or screening time lasted 8 minutes per call. A total of 41,123 (36.7%) callers were identified as being at risk of poor outcomes; they received recommendations to seek health care assistance at a local emergency medical unit.

The Telecoronavirus program recruited 1396 medical students as volunteers from 12 public and private medical schools across the state of Bahia. Among these, 1119 (80.2%) students were in their final two years of medical studies, and 921 (66%) were female. The supervisor group comprised 133 volunteer physicians, including residents and medical doctors.

The majority of volunteers reported that their experience bridged together the practical experiences of telescreening and provided them with learning resources, such as short videos and scientific texts, while also helping them acquire knowledge about COVID-19 by summarizing emerging scientific evidence (Table 1).

The participating physicians and students reported that the service greatly contributed to the development of telemedicine- or telehealth-related skills, as indicated by scores of 9.0 and 8.8, respectively, on a scale from 0 to 10. Furthermore, 99.6% (802/805) of volunteers indicated they would be interested in performing volunteer work in the health area again (Table 1).

Volunteers reported that the Telecoronavirus program was well-organized and that it operated smoothly. They found the screening algorithms to be clear and easy to use, and they positively evaluated the remote supervision method employed. The volunteers’ perception was that patients followed the medical advice offered and adhered to the general guidance provided regarding hygiene and social distancing (Table 1).

**Table 1:** Evaluations from physician and student volunteers regarding the development of telemedicine- or telehealth-related skills while using the Telecoronavirus program (Bahia, Brazil).

Question topics	Evaluation scores (range 0-10)	
	Students (n=805)	Physicians (n=55)
Remote supervision of telehealth service, mean (SD)	8.63 (1.40)	—^a^
Flowchart quality, mean (SD)	9.34 (1.07)	9.02 (1.05)
Perception that patients followed recommendations, mean (SD)	8.63 (1.00)	—
Social impact of Telecoronavirus service, mean (SD)	9.16 (1.01)	9.25 (1.55)
Learning about COVID-19, mean (SD)	8.84 (1.34)	—
Telemedicine skills, mean (SD)	9.09 (1.07)	8.80 (1.64)
Telehealth skills, mean (SD)	9.33 (0.95)	—
Social commitment and citizenship, positive answer, n (%)	743 (92)	46 (84)
Sense of accomplishment, positive answer, n (%)	773 (96)	52 (95)

^a^Not applicable, as these questions were addressed only to medical students.

## Discussion

### Principal Findings

Our experience revealed that the remote supervision of medical students by physicians, supported by clear guidelines, enabled us to offer a high-quality telescreening service in the face of scarce medical staff and materials in a pandemic scenario requiring social distancing. The Telecoronavirus program proved to be a simple, low-cost (approximate cost US $0.36 per user), and far-reaching strategy (serving 82% of the state’s municipalities), which can be readily applied in the context of other situations where health services are in high demand. Our experience shows that establishing partnerships between governmental and academic actors can provide valuable and innovative solutions for handling public health emergencies.

COVID-19 is predicted to remain a public health concern in the near future and will continue to impose challenges to both health care practices and medical training. A combination of remote tools with medical supervision can be applied to provide practical experience while still maintaining social distancing measures. The experimental method adopted effectively allowed medical students to contribute to mitigating the impact of the pandemic in conditions that required workforce reductions.

Considering that almost 70,000 users were advised to stay at home and received guidance on how to mitigate disease transmission, the Telecoronavirus program may have also contributed to a reduction in the spread of the virus by preventing the unnecessary circulation of mild cases. Modeling-based studies have provided evidence that undocumented infections (including mild cases) can be the source of almost 80% of reported COVID-19 cases [15]. Our approach was capable of leveraging technical aspects of medical knowledge, the potential of telemedicine services, and assistance provided by well-trained volunteers to provide free-of-charge health guidance to the state’s population. This citizenship-promoting activity brought medical students closer to the real-life activities and challenges faced by the public health system in times of distress and vulnerability.

During the SARS epidemic of 2002, several provinces in China and Taiwan maintained hotline consultation services that provided medical counseling, including instructions on infection prevention and tracking of suspected cases.[16]. The main disadvantage of these services was the reallocation of health professionals to telescreening activities, reducing the number of regular medical personnel. As the present initiative recruited medical students who were supervised by physicians who were not working at the frontline, it was not necessary to reallocate any frontline medical workers.

Similar initiatives involving medical student engagement in an attempt to mitigate the COVID-19 pandemic have been previously described [17,18]. Medical students from the Ochsner Clinical School (New Orleans, LA, USA) participated in a hotline service, considerably reducing caller wait times and providing support for a symptom tracking program [17]. A similar project was developed in the state of Nevada (USA), wherein medical students participated in a telescreening service to assist underserved rural populations [18]. In both cases, students gathered in a physical environment on their university campus to carry out telescreening activities. In contrast, in the Telecoronavirus program, patient calls were redirected to medical students’ mobile phones. Supervision was performed remotely, thereby eliminating the need for transportation and public gatherings, which further reduced the potential risk of SARS-CoV-2 infection among students.

In addition to technical aspects, the personal and social benefits of performing volunteer activities and contributing to the mitigation of the burden imposed by the pandemic in their respective communities may have raised social consciousness among the participating students and physicians.

As a strategy to advise patients suspected of COVID-19, telescreening conducted by medical students offers several advantages compared to automated online screening services: (1) the execution of a screening protocol by a qualified group minimizes interpretation bias and can provide rapid responses in unexpected situations, (2) users are not required to possess a minimum level of formal education level or be digitally literate in order to access the service, and (3) no internet access is required [19]. These points are particularly important in low- and middle-income countries, such as Brazil, where despite recent improvements, a digital divide remains throughout the country’s vast territory, as well as across specific age groups, particularly among the elderly [20]. Additionally, the fact that health care team members provide a more humanized service increases adherence to social isolation guidelines and recommendations to seek medical care.

### Limitations

Our work presented some limitations due to the challenging conditions imposed by the real-time mobilization during the pandemic. The screening algorithm specifically targeted respiratory diseases. However, in addition to the COVID-19 pandemic, the state of Bahia faced two other concomitant outbreaks of acute viral infections: dengue and chikungunya. The absence of specific flowchart directions for suspected arbovirus infections resulted in the systematic guidance of patients to seek medical care.

It was unfortunately not possible to obtain formalized feedback from users, who could have provided valuable information to improve the quality of the service. However, some users did call back to notify volunteers that their symptoms had improved, and some even made a point to thank the volunteers for their service, according to reports by volunteers via the messaging application to their workgroups.

Although psychological assistance was made available to all project volunteers, our flowchart did not include protocols to address users’ mental distress. To mitigate this deficiency, we provided practical guides and video lessons designed to help volunteers navigate situations in which users were experiencing psychological duress, as applicable within the context of telehealth services.

Several patients reported an inability to self-isolate at home due to social and economic conditions. In the final month of the program’s operation, this issue was partially mitigated through referrals of patients to government shelters made available in the state’s capital.

### Conclusions

Remote tools for the early detection of respiratory infection outbreaks, provided at no cost to users, will be of paramount relevance to the strengthening and expansion of classical epidemiological surveillance activities.

Additionally, establishing connections between the provision of health care services and medical student education and experiential learning may also apply to patients with other illnesses, both acute and chronic, reducing the need for face-to-face consultations and the risk of infection transmission among patients, healthcare team members, and administrative staff.

## Appendix

Multimedia Appendix 1 Telecoronavirus flowchart (complete version) used by volunteers for screening possible COVID-19 cases (Bahia, Brazil).

Multimedia Appendix 2 Online post-project survey (Telecoronavirus program, Bahia, Brazil).
