# Supplementary figures and images for: Bridging Learning in Medicine and Citizenship During the COVID-19 Pandemic: A Telehealth-Based Case Study
Source: JMIR Public Health Surveill. 2021 Mar 4;7(3):e24795. doi: 10.2196/24795 (PMC7935247; doi:10.2196/24795)

**Tele Coronavirus**  
**Version 20**  
**Date: 31/07/2020**

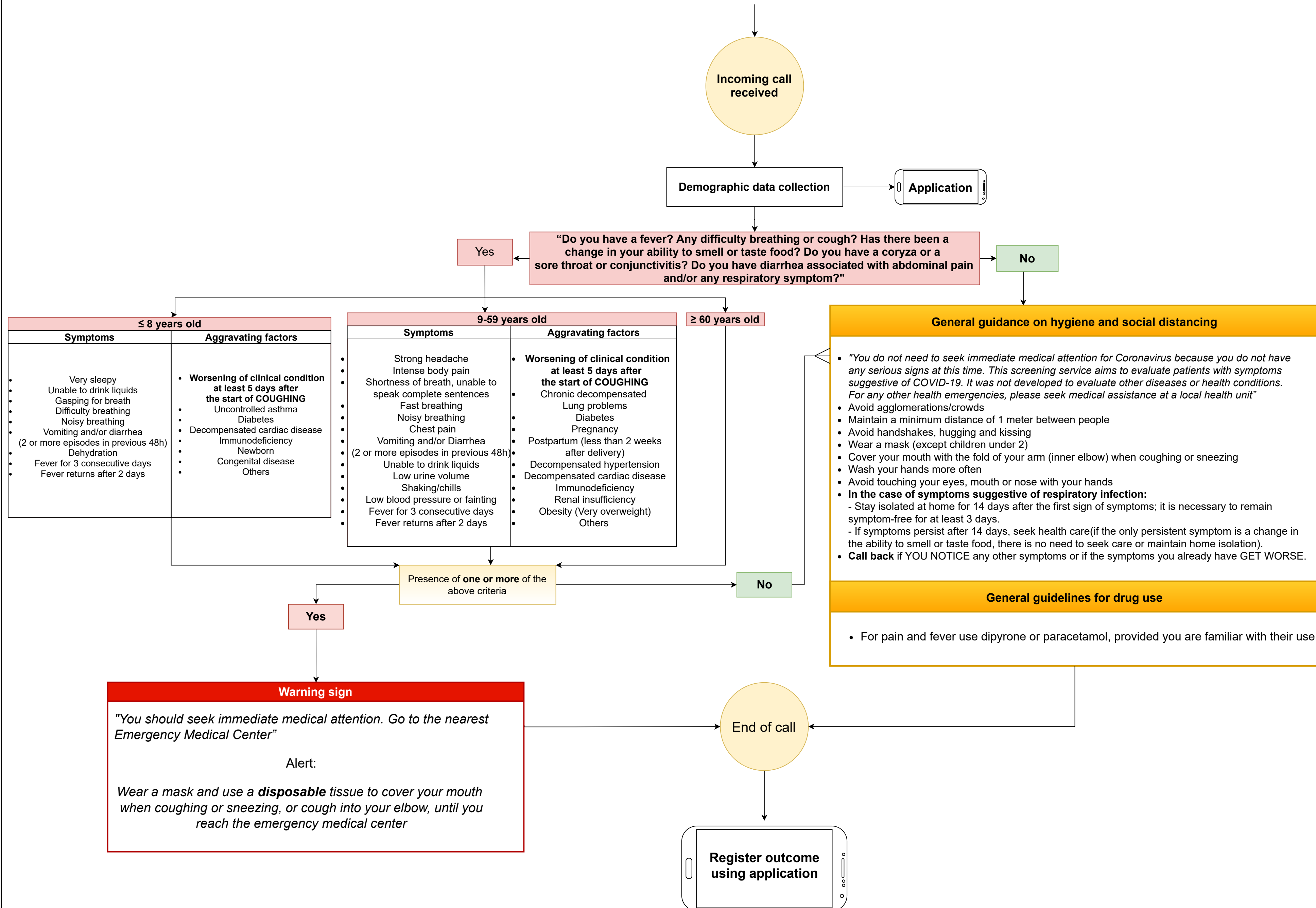

Supplement: Multimedia Appendix 1 [file publichealth_v7i3e24795_app1.pdf]
