# Supplementary material for: Bridging Learning in Medicine and Citizenship During the COVID-19 Pandemic: A Telehealth-Based Case Study
Source: JMIR Public Health Surveill. 2021 Mar 4;7(3):e24795. doi: 10.2196/24795 (PMC7935247; doi:10.2196/24795)
Supplement: Multimedia Appendix 2 [file publichealth_v7i3e24795_app2.docx]

Participant survey sent to volunteers upon program termination. Questions with a scale from 1 to 10 correspond to very poor (1) to excellent (10).

1. In which year of your medical studies were you registered when you started volunteering for the Tele-Coronavirus program?
   1. 4/5/6
2. Did you have any previous experience with telescreening?
   1. Yes/No
3. Have you previously done any voluntary work?
   1. Yes/No
4. Overall, how would you rate your experience as a volunteer in the TeleCoronavirus program?
   1. 1 to 10
5. Would you volunteer again to work in the health area?
   1. Yes/No
6. Did you find the content of the mini-video lessons recorded by instructors from various institutions useful?
   1. Yes/No
7. How would you rate the model of supervision employed via messaging app?
   1. 1 to 10
8. How would you evaluate the flowchart used for telescreening?
   1. 1 to 10
9. What is your general perception regarding the callers’ acceptance of the orientation provided via telescreening?
   1. 1 to 10
10. In your opinion, how would you rate the impact of the Telecoronavirus service on the community?
    1. 1 to 10
11. How important was your participation in the Telecoronavirus program with respect to learning about telemedicine in general?
    1. 1 to 10
12. How important was your participation in the Telecoronavirus program with regard to developing telescreening skills?
    1. 1 to 10
13. How important was your participation in the Telecoronavirus program in terms of learning about COVID-19?
    1. 1 to 10
14. Regarding your participation in Telecoronavirus, check the options listed below that you consider relevant to your overall evaluation of the program. ╪
    1. Satisfaction from contributing my time, skills and knowledge to mitigate the effects of the pandemic.
    2. Opportunity to develop new skills in telemedicine.
    3. Social commitment and citizenship.
    4. Contribution to my curriculum vitae (resumé) with skills, initiative and availability
    5. Practical experience in "real world" healthcare
    6. Expansion of professional network and making contacts with colleagues, doctors, professors, etc.

╪Each item checked was considered as a positive response.
